# MHC and Evolution in Teleosts

**DOI:** 10.3390/biology5010006

**Published:** 2016-01-19

**Authors:** Unni Grimholt

**Affiliations:** Department of Virology, Norwegian Veterinary Institute, Ullevaalsveien 68, Oslo N-0106, Norway; unni.grimholt@vetinst.no; Tel.: +47-2321-6376

**Keywords:** teleost, rayfinned fish, Major histocompatibility complex, MHC class I, MHC class II, phylogeny, evolution

## Abstract

Major histocompatibility complex (MHC) molecules are key players in initiating immune responses towards invading pathogens. Both MHC class I and class II genes are present in teleosts, and, using phylogenetic clustering, sequences from both classes have been classified into various lineages. The polymorphic and classical MHC class I and class II gene sequences belong to the U and A lineages, respectively. The remaining class I and class II lineages contain nonclassical gene sequences that, despite their non-orthologous nature, may still hold functions similar to their mammalian nonclassical counterparts. However, the fact that several of these nonclassical lineages are only present in some teleost species is puzzling and questions their functional importance. The number of genes within each lineage greatly varies between teleost species. At least some gene expansions seem reasonable, such as the huge MHC class I expansion in Atlantic cod that most likely compensates for the lack of MHC class II and CD4. The evolutionary trigger for similar MHC class I expansions in tilapia, for example, which has a functional MHC class II, is not so apparent. Future studies will provide us with a more detailed understanding in particular of nonclassical MHC gene functions.

## 1. Introduction

Originally, the histocompatibility antigen (or the H2-locus) was defined as a gene with a major effect on tissue rejection in mice. Later, when it became obvious that there were several closely-linked genes that influenced rejection, the region was defined as the major histocompatibility complex, or MHC in short. These and similar discoveries in humans provided George Snell, Baruj Benacerraf, and Jean Dausset with a Nobel Prize in medicine in 1980 and paved the way for successful tissue and organ transplantation. It also opened the field of MHC and evolution.

The core human MHC or human leucocyte antigen (HLA) region, as we know it today, is a gene dense four-megabase region with 224 identified genes. It is historically divided into MHC class I, MHC class II, and MHC class III regions. The MHC class I (MHCI) region encompasses genes for the three classical MHC class I loci *HLA-A*, *HLA-B*, and *HLA-C* molecules in addition to genes for the nonclassical class I molecules *MICA*, *MICB*, *HLA-E*, *HLA-F*, and *HLA-G*. To be defined as classical MHCI, the molecules must bind peptides, be expressed in a wide range of tissues or cell types, and exist in many allelic variants per locus. This variability between alleles is primarily located within the peptide binding domains, providing a population with a set of molecules that can bind and present a wide range of different peptide motifs. Genes for the MHCI-specific proteasome and transporter are located in between the MHC class II genes. The MHC class II (MHCII) region encompasses genes for three different classical MHCII molecules, denoted HLA-DR, HLA-DP and HLA-DQ in addition to two nonclassical molecules denoted HLA-DM and HLA-DO, each encoded by one alpha and one beta gene. The classical definition for class II includes binding of peptides, high polymorphism, and expression on antigen presenting cells. The MHC class III region includes many other immune genes, such as complement factors C2 and C4, heat shock proteins, and tumor necrosis factor.

Classical MHC class I molecules, combining an alpha and a beta2-microglobulin chain, are present on most cells and bind and present endogenously-derived peptides to CD8+ T-cells. The alpha chain consists of three domains where the alpha 1 and alpha 2 domains provide a size-restricting groove for peptides 8–11 amino acids (aa) long. In humans, the three classical I genes, called *HLA-A*, *HLA-B*, and *HLA-C*, are some of the most polymorphic genes known to date with 2313, 3011, and 1985 amino acid alleles from each of the three loci, respectively [1]. This extreme polymorphism is assumed to be driven by coevolution with rapidly evolving pathogens. The allelic variation is primarily found in the two peptide-binding domains alpha 1 and alpha 2. After synthesis, the MHCI molecule is stabilized by tapasin and other chaperones in the ER prior to loading with peptides. In general, self and, for example, viral, proteins residing in the cytosol are degraded by a proteasome complex including the inducible sub-components PSMB8-10 and are then transported into the ER through the MHC-linked transporter TAP1-TAP2. Presentation of endogenous peptides on the surface provides the NK-receptor with an "all-clear" signal while MHC-exogenously-derived peptide complexes induce a cytotoxic response through interaction with T cell receptors on CD8+ T cells.

In addition to the three human classical genes, there are also nonclassical genes called *HLA-E*, *HLA-F*, and *HLA-G*. These genes are less polymorphic with 6, 4, and 16 alleles, respectively, and generally have more restricted expression patterns. HLA-E molecules primarily bind leader sequences from classical MHC molecules and interact with NK receptors [2,3], but may also bind other ligands and interact with alpha/beta T-cell receptors on CD8+ T cells [4]. HLA-F molecules do not bind peptides, but instead interact with peptide-devoid MHCI molecules or so-called open conformers (OC) [5,6,7]. This association between HLA-F and OCs has been suggested as a cross-presentation pathway for capture and loading of MHCI molecules with exogenous peptides. HLA-G molecules bind a limited repertoire of peptides, are primarily expressed at the fetal-maternal interface, and are thus thought to play a role in protecting the fetus from maternal immune rejection [8].

In humans, there are several other molecules that all share the MHCI alpha 1 and alpha 2 domain structure of two helices lining a beta-pleated sheet with classical MHCI [8]. Some of these molecules have a three extracellular domain alpha chain and associate with beta2-microglobulin molecules, such as CD1, FCGRT (FcRn), and HFE. Others, such as ZAG (AZGP1) and MICA/B, have retained the three-domain alpha-chain structure, but lost the ability to bind beta2-microglobulin. Yet others, such as ULBPs and PROCR, have lost the alpha 3 domain and thus also the ability to bind beta2-microglobulin. Functionally, these molecules are quite different from classical MHCI molecules, where, for instance, MICA/MICB as well as the ULBPs interacts with the NK receptor NKG2D. Human CD1a-d molecules bind lipids acquired in endosomal compartments and present these to T cells [8,9,10]. CD1a and CD1d even associate with Invariant chain, a MHC class II assistant, for intracellular transport [11]. CD1e does not interact with T cells, but assists in the lipid-processing and loading of other CD1 molecules. Endothelial Protein C Receptor (PROCR) is best known for its function as an anticoagulant, by directly binding to and activating protein C to promote anticlotting [8]. More recently, a broader view of PROCR function is emerging with additional roles also in inflammation and apoptosis [12]. Other MHCI-like molecules include AZGP1, which binds hydrophobic ligands and participates in lipid homeostasis, FcRn, which is involved in uptake and transportation of maternal immunoglobulin to unborn or weening offspring, HFE, a transferrin-binding protein that regulates iron homeostasis, and MR1, which is shown to present microbial vitamin B metabolites to mucosal-associated invariant T (MAIT) cells [8,13,14].

The MHC class II (MHCII) molecule consists of an alpha and a beta chain, each composed of two domains. It primarily resides on the surface of antigen-presenting cells and binds and presents peptides derived from exogenously-derived proteins to CD4+ T cells. The 18–20 amino acid long peptides are bound in an open groove provided by the alpha 1 and beta 1 domains. These peptide-MHCII complexes then interact with CD4+ T helper cells, thus inducing downstream responses such as localized inflammation and swelling due to recruitment of phagocytes, or activation of B cells, for instance. In humans, the classical MHCII molecules are encoded by the *HLA-DPA*/*-DPB*, *HLA-DQA*/*-DQB*, and *HLA-DRA*/*-DRB* genes that currently count 501, 627, and 1404 amino acid alpha and beta alleles, respectively [1]. In humans, the beta loci are by far more polymorphic than the alpha loci. Newly synthesized molecules are bound to invariant chain in the endoplasmic reticulum, which provides a transport signal to late endosomes for peptide loading (reviewed in [15]). Peptide loading/editing is assisted by a nonclassical MHCII molecule called HLA-DM, also in the format of a combined alpha and beta chain encoded by the *HLA-DMA* and *HLA-DMB* loci. Another nonclassical class II molecule, *i.e.*, HLA-DO, encoded by the *HLA-DOA* and *HLA-DOB* genes, acts as a regulator of HLA-DM function. The nonclassical HLA-DM and HLA-DO genes are far less polymorphic with 11 and 8 amino acid alleles counted so far.

Prior to the discovery of MHC molecules in carp in 1990 [16], the teleost immune system was expected to be somewhat primitive as compared to its mammalian counterparts. Since then our view has gradually expanded, although the view still prevails that the innate immune system is more important in teleosts than the adaptive immune system. However, considering available data, we are still at the very beginning of understanding the complexity of the teleost MHC.

## 2. Teleost MHC

A systematic MHC nomenclature for all species was first proposed by Klein *et al.* [17], where MHC sequences from various species should be named Mhc followed by the two first letters of Latin names e.g., MhcSasa for a sequence from *Salmo salar*. This prefix should then be followed by a letter describing the class, the locus and finally the subclass. With D for duo or second reserved for MHC class II (see below), teleost MHC class I nomenclature used U for uno for the first identified MHC class I sequences. Nomenclature for teleost MHC class I genes have since evolved, with U defining sequences belonging to the U lineage, while new lineages have been named according to the species they were discovered in or other specifics related to the first identified sequences of that lineage.

**Figure 1:**
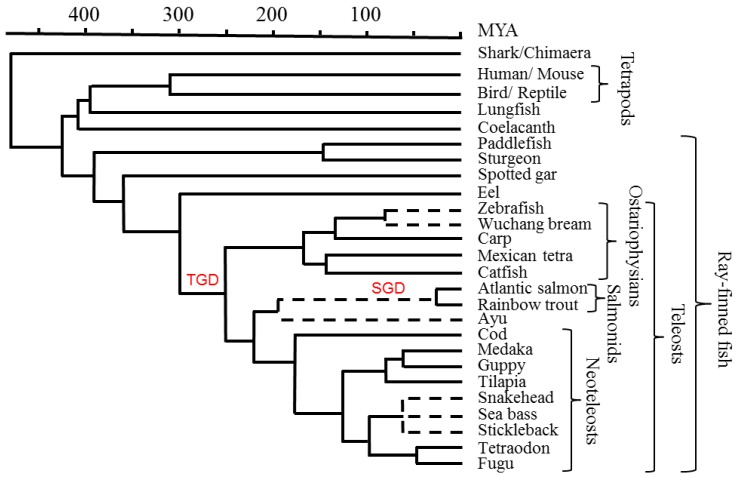
Phylogeny of relevant species. A timescale is depicted in millions of years ago (MYA). Dotted lines relate to phylogenetic branch knots where the referenced literature was not informative on the absolute time of the event. Whole genome duplication events in a teleost ancestor (TGD), and early in the salmonid lineage (SGD), are indicated in red font. Figure modified from [18,19].

The data presented in this review mainly originate from the comprehensive teleost MHC class I and MHC class II studies performed by Dijkstra *et al.* [18] and Grimholt *et al.* [19]. These data primarily resulted from analyses done in species with sequenced genomes, including fugu, tetraodon, tilapia, platyfish, medaka, cod, Atlantic salmon, zebrafish, and Mexican tetra. See Figure 1 for phylogenetic relationships between the different species. Spotted gar was included as the only primitive bony fish with a sequenced genome, enabling phylogenetic tracing of the lineages.

Although this review aims at summarizing the main outline of teleost MHC, salmonids are given a particular focus. This is mainly due to the fact that Atlantic salmon and rainbow trout are two species where there is a well-defined segregation between classical *versus* nonclassical MHC genes, where defined allelic variants belonging to the classical MHC class I and class II genes can be found in an easily-accessible database [1]. Emerging data from other teleost species such as medaka and zebrafish suggest that the picture drawn in salmonids is mostly representative for that found in other teleosts species.

### 2.1. Teleost MHC Class I

Currently, the five different MHC class I U, Z, S, L, and P lineages have been identified in teleosts based on phylogenetic clustering [19]. A more detailed summary of each lineage is presented below.

#### 2.1.1. The U Lineage

Only a few species have been studied in sufficient detail to enable definition of classical and nonclassical U lineage genes. The first classical teleost MHC class I sequence was reported in Atlantic salmon by Grimholt and co-workers in 1993 [20], and the gene, later denoted *SasaUBA*, was shown to exist in a variety of allelic variants [21]. Classical-type MHC class I molecules have since been substantiated in medaka [22], rainbow trout [23], and pink salmon [24]. Definition wise, the UBA locus in Atlantic salmon and rainbow trout and the *UAA* and *UBA* loci in medaka are all representatives of classical loci, each containing a wide range of alleles and being expressed in most tissues [19,21,22,25]. There are currently 44 and 50 registered alleles for the classical Atlantic salmon and rainbow trout UBA gene respectively [1]. In zebrafish, potential classical genes denoted UBA through UFA residing within an orthologue major MHCI region have been described [26,27], with haplotypes containing one to three classical-like genes [28]. The polymorphic content of each of these genes is still undefined, postponing their definition as true classical genes. Haplotype variation has also been shown for the medaka MHCI region, but here the polymorphic nature has been verified for the classical UAA and UBA genes while other U lineage genes residing within this region have been defined as nonclassical with low polymorphism [22,29].

**Figure 2:**
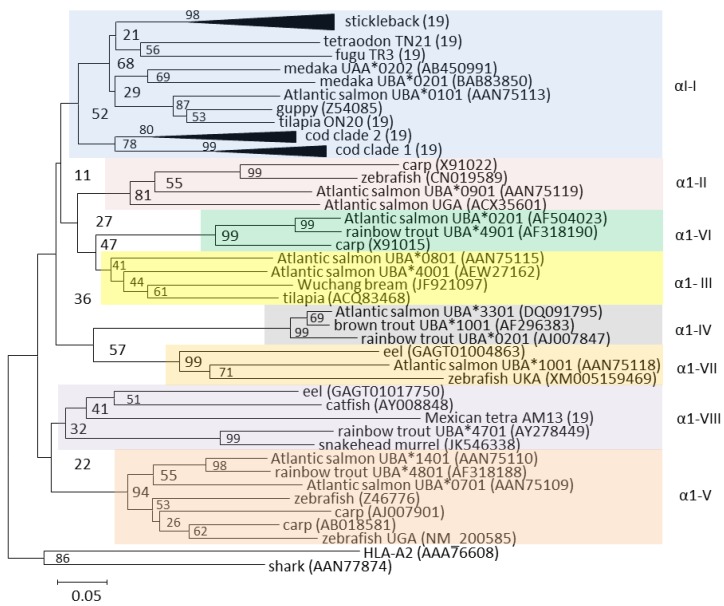
Phylogeny of selected U lineage alpha 1 domain amino acid sequences from Grimholt *et al.* [19]. The eight U lineage alpha 1 domain lineages are shown as shaded boxes with Roman numbers outside each lineage cluster. The black triangles shown for stickleback and the two cod clades are collapsed clusters of 25 and 10 sequences each, respectively. GenBank sequence references are shown using parentheses, while the remaining sequences and sequence references can be found in [19]. The tree is made using Neighbor-joining P-distance and pairwise deletions. The tree is drawn to scale, with branch lengths representing the number of amino acid substitutions per site (see scale bar). Bootstrap values in percentage from 1000 trials are shown. The tree is rooted using the human HLA-A2 and shark sequences.

An intriguing aspect of teleost U lineage genes is the existence of ancient alpha 1 and to a lesser degree alpha 2 domain lineages shared between distantly related species [19,23,30,31,32] (Figure 2). A large intron between the alpha 1 and alpha 2 domains is assumed to enable intra-locus recombination, allowing distinct alpha 1 domains to be shuffled between alpha 2 and downstream sequences. Several of these alpha 1 domain lineages can be traced back to a common ancestor of eel and the bulk of teleosts (Figure 2). As opposed to salmonids, with at least eight different alpha 1 domain lineages, the α1-I lineage seems dominant in neoteleosts [19]. Cod and stickleback have expanded this alpha 1 domain lineage while they seem to have lost the other seven α1 lineages found in salmonids. In Atlantic cod, this α1-I lineage has evolved into two distinct clades, potentially to compensate for the loss of its MHCII machinery [33]. Exactly how this dual nature is linked to presentation of exogenous peptides remains unknown. However, sequences from one of the clades contain endosomal sorting signal, hinting at cross-presentation through acquisition of exogenous peptides. Stickleback does not display this dual nature of its alpha 1 α1-I domains, possibly due to the continued presence of functional MHC class II molecules [18].

**Figure 3:**
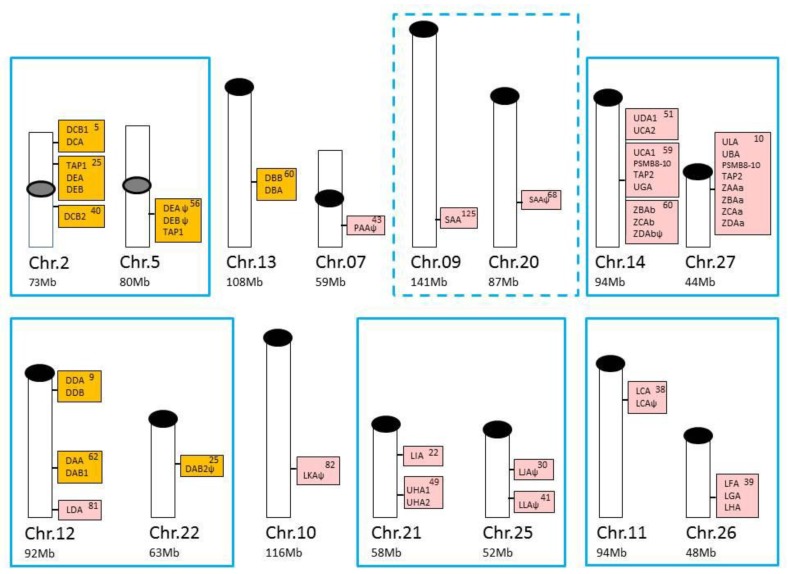
Chromosomal locations of Atlantic salmon major histocompatibility complex (MHC) class I and class II genes, in addition to PSMB8-10 and TAP1/2 genes based on data from [18,19]. Atlantic salmon MHC class I genes are shown with pink shading, while class II genes are shown with orange shading on individual chromosomes (NCBI genomes, GCA_000233375.4). Total size of each chromosome (Chr.) is shown below in megabases (Mb), while location of MHC genes are shown within each box. Centromeres and telomeres are shown with grey (tentative position) or black oval shapes. MHC-containing chromosomes known to be homologous or share regional homology due to the unique salmonid Ss4R WGD event [39,40] are shown with cyan boxes where the presumed shared homology between Chr.09 and 20 is tentative and thus shown using a dotted line.

Additional salmonid U lineage loci, defined as UBA, UCA, UDA, UEA, UFA, UGA, UHA, and ULA [25,30,34], mainly reside within two duplicate MHCI regions [35,36,37] that originated due to a unique salmonid whole genome duplication (WGD) event that occurred approximately 95 million years ago, referred to as the Ss4R genome duplication [38]. The classical UBA locus resides in what is now denoted the Ia region, while the Ib region contains the majority of nonclassical U lineage genes. An illustration of the chromosomal location of all Atlantic salmon MHC class I and class II genes is shown in Figure 3.

The nonclassical salmonid UCA, UDA, UEA, UFA, ULA, UGA, and UHA genes have mostly retained the conserved residues known to bind peptide termini, have limited polymorphism, and mostly a more restricted expression pattern [34,35,36,37,41]. For nonclassical U lineage sequences from other species, some display the conserved residues known to bind peptide termini in mammalian MHCI sequences, while others display less conservation of these residues [19]. In medaka, the two nonclassical U lineage genes *orlaUDA* and *orlaUEA* reside in the MHCI region, while the four genes *orlaUCA*, *orlaUGA*, *orlaUHA*, and *orlaUIA* reside elsewhere [31]. Although we have no data suggesting the function of these nonclassical U lineage genes, one may speculate that they for instance perform functions analogous to the human HLA-E or HLA-F molecules.

In salmonids and medaka, the number of classical U lineage loci is one and two, respectively, while the number of nonclassical U lineage loci is somewhat higher. However, there are species that have chosen another approach. Atlantic cod has approximately 100 U lineage genes with at least 83 unique expressed sequences from one individual [33,42]. This gene expansion has been suggested as a compensation for the lack of MHC class II in cod [33] and exemplifies a situation where the traditional terms of classical *versus* nonclassical loci no longer apply. The term “polygenic variability” has thus been suggested to describe this unique mode of generating and maintaining MHC variation [19]. Although not as extreme, 45 and 29 MHC class I U lineage genes or gene fragments have been reported in tilapia and stickleback, respectively [19,43]. Both tilapia and stickleback still have functional MHC class II molecules, so the trigger for expanding the U lineage is not so apparent.

#### 2.1.2. The Z Lineage

The first MHC class I and class II sequences to be identified in teleosts were from cyprinids [16]. These MHC class I gene sequences were expressed and defined as ZA–ZD [44], and were shown to cluster closest to nonclassical MHCI sequences of a distinct lineage, defined as the Z lineage [36,45,46,47].

**Figure 4:**
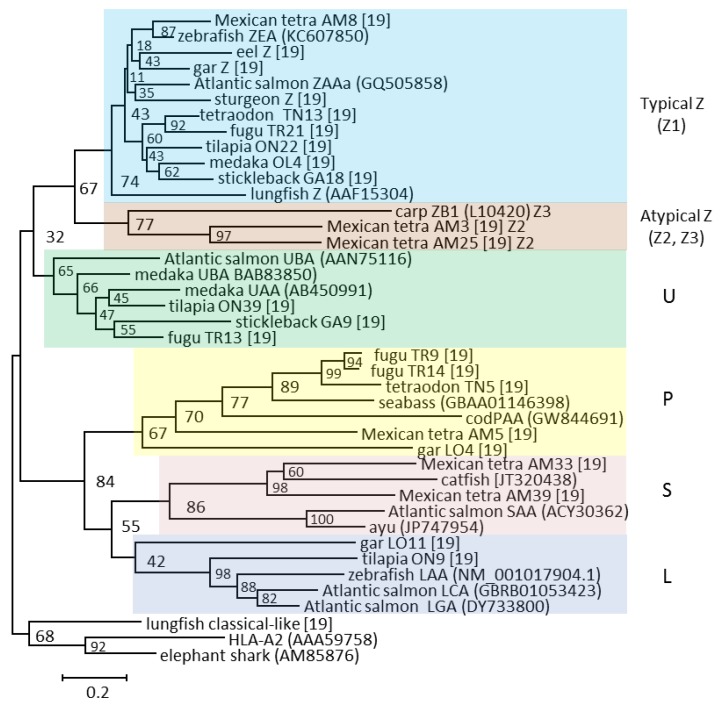
Phylogeny of selected alpha 1 domain amino acid sequences from the five defined lineages is shown using different colored shading. The tree is based on a simplified manually curated alignment from Grimholt *et al.* [19]. The tree is drawn to scale, with branch lengths representing the number of amino acid substitutions per site (see scale bar). Bootstrap values in percentage based on 1000 trials are shown on each internal node. The atypical Z lineage definitions Z2 and Z3 are shown behind the sequence names. GenBank sequence references are in parentheses while the remaining sequences can be found in [19].

The ZA–ZD genes differed significantly from U and Z lineage sequences identified in other species, and were until recently considered unique to cyprinids [16,44]. However, we found that also Mexican tetra, another Ostariophysi, have similar although not identical Z lineage sequences alongside sequences of a more typical Z nature [19]. Thus, Z lineage sequences are now defined as either typical, belonging to the Z1 lineage, or atypical, belonging to the Z2 (Mexican tetra) or Z3 (cyprinids) sub-lineages (Figure 4). As opposed to typical Z lineage sequences being likely peptide binders, the atypical sequences lack many of these peptide-binding residues and most likely do not bind peptides [19].

**Figure 5:**
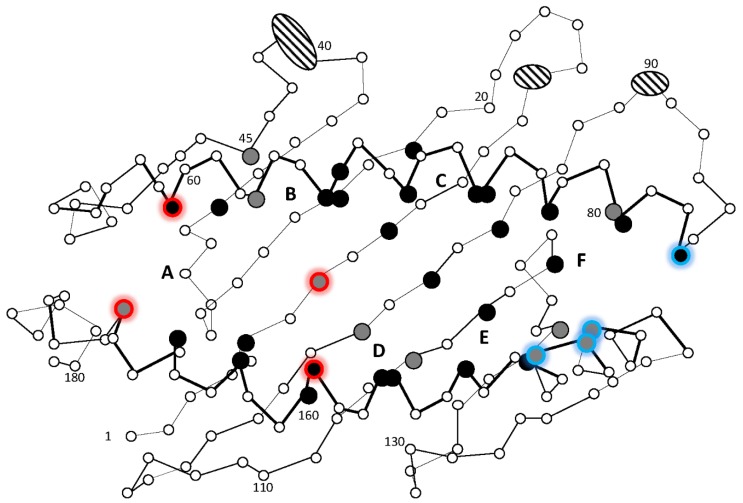
Schematic presentation of the human HLA-A2 peptide binding groove defined by the alpha 1 and alpha 2 domains. The 37 residue positions in HLA-A2 known to contribute to the six A through F pockets [48,49] are enlarged and colored black when completely conserved in the aligned teleost Z1 sub-lineage sequences, while grey represents teleost sequence identity of 90%–99.7%. Residues anchoring peptide N terminal and C terminal ends are shown using red and blue glow respectively. Ellipses cover those HLA-A2 residues that, according to our sequence alignment, have few or no matching residues in the aligned teleost sequences. The figure is based on 31 Z1 lineage sequences from eight teleosts, where sequences, alignment, and original figure can be found in [19].

The number of Z lineage genes range from 18 genes in Mexican tetra to one gene in stickleback, tetraodon, and cod. At least one typical Z lineage gene is expressed in all studied species, suggestive of a vital biological function. A remarkable feature of the typical Z lineage sequences is a complete conservation of the residue positions contributing to the peptide-binding groove when aligned against the human HLA-A2 sequence. Typical Z lineage sequences from all investigated teleosts have an almost complete conservation of the 37 residue positions known to provide the HLA-A2 molecule with the six A through F peptide-binding pockets (Figure 5).

This almost complete sequence conservation suggests that all these typical molecules have a common but as yet unknown peptide ligand. Sequences belonging to this typical Z lineage have also been found in the primitive ray-finned fishes eel, spotted gar and sturgeon, in addition to a MHCI sequence from lungfish, all displaying the same conserved motif. Thus, the unique and as yet unknown function of the typical Z lineage has existed for more than 450 million years and most likely represents an essential biological function. Future studies will reveal if this lineage for instance performs a similar function as the human MR1 molecule, presenting vitamin B metabolites to Mucosal-associated invariant T (MAIT) cells.

In Atlantic salmon, the six Z lineage genes reside in the two duplicate MHC Ia and Ib regions on chromosome 27 and 14, respectively (Figure 3). Some of the Atlantic salmon genes have quite distinct transcription profiles with *SasaZCAa* dominating in gill, *SasaZDAa* dominating in head, kidney, and spleen, while *SasaZBAb* and *SasaZCAb* dominating in gut [19]. This differential expression pattern, or sub-functionalization of duplicates, is also found for some of the twelve zebrafish Z lineage genes [50], where haplotypic variation seems to add an additional level of diversification to the Z gene repertoire across individual zebrafish.

#### 2.1.3. The S Lineage

S lineage sequences were first identified in salmonids and were named UAA by Shum *et al.* [51]. Later, when these sequences were also found in catfish belonging to Siluriformes, the sequences were redefined to SAA, reflecting their origin, but also underlining their phylogenetic separation from U lineage sequences [35]. Such S lineage sequences have now also been found in Mexican tetra (an Ostariophysi) as well as ayu, an Osmeridae related to salmonids (Figure 4) [19].

The peptide anchoring residues typical for the peptide-binding classical MHCI sequences are not conserved in S lineage sequences, suggesting they have non-peptide or no ligands. Some S lineage sequences have additional cysteines in their alpha 1 and alpha 2 domains, but none conserved between salmonids and ostariophysians. A common feature for all S lineage sequences is a very short cytoplasmic tail, which may indicate a much longer half-life at the cell surface, similar to HLA-G [52]. In Atlantic salmon, the single S lineage gene displays a rather low expression in immunologically important tissues, and an almost silence in non-immunological tissues [19], providing no clues as to the functional relevance of this lineage.

#### 2.1.4. The L Lineage

The L lineage was first found in salmonids and zebrafish by Dijkstra *et al.* [53] and was named L due to an unusual linkage of two L lineage genes with an MHC class II gene in zebrafish. Presence of L lineage genes in zebrafish has later been expanded upon by Dirscherl *et al.* [54], and in other teleost and ray-finned species by Grimholt *et al.* [19]. Apart from the ten and 16 loci found in Atlantic salmon and zebrafish respectively, two genes have been found in Mexican tetra and spotted gar, while tilapia had one L lineage gene. The two Mexican tetra genes are both pseudogenes. Phylogenetic clustering of L lineage sequences in relation to the other lineages is presented in Figure 4.

A unique feature of some L lineage genes is the loss of introns [19,53]. In trout, the *LAA* gene has the usual introns separating the alpha 1, alpha 2, and alpha 3 domains, while the trout *LBA*, *LCA*, and *LDA* genes lack all of these introns. The tilapia gene has an intermediate gene organization where it has lost the intron between the alpha 1 and alpha 2 domains, but retained the intron between the alpha 2 and alpha 3 domains. Despite loss of introns, at least six of the L lineage genes in Atlantic salmon are expressed, although at fairly low levels [19].

L lineage sequences do not contain the typical peptide-binding residues, and thus likely either bind non-peptides or no ligands. However, the fact that L lineage sequences have the overall highest hydrophobicity of all five lineages in the ligand binding domains alpha 1 and alpha 2 makes this lineage the best candidate for binding and presentation of (glyco)lipids similar to the human CD1 molecules.

#### 2.1.5. The P Lineage

The fifth and final MHC class I lineage was designated P due to its discovery in pufferfishes (Figure 4) [19]. P lineage pseudogenes are found in salmonids and sablefish, but bona fide genes are found in Mexican tetra, cod, tetraodon, fugu, seabass, and spotted gar. This lineage has undergone a drastic expansion in fugu, with 24 loci where at least eight genes contain alpha 1 through alpha 3 domains. Allelic variation and expression patterns of these fugu genes are currently unknown.

P lineage sequences do not contain the residues expected for peptide binding, so they most likely bind non-peptides or no ligands. The P lineage sequences display two additional cysteines in the alpha 1 domain in similar positions as seen for some alpha 1 domain U lineage sequences [31]. Looking at these residue positions in three dimensions aligned against the human HLA-A2 molecule, these cysteines reside in close proximity and could easily form a bond between the beta sheet and the alpha helix, thus influencing the shape and flexibility of the potential ligand-binding groove.

In Atlantic cod, the single P lineage gene was not expressed in immunologically-important tissues such as spleen, hind gut, and head kidney underlining its nonclassical nature. However, both cod as well as tetraodon displayed P lineage expression in brain, suggesting that this gene may be involved in brain development and function similar to the emerging roles of mammalian nonclassical MHCI molecules in this organ [55,56,57].

### 2.2. Teleost MHC Class II

The teleost MHCII nomenclature uses D or duo for the second class, followed by a letter referring to family, and finally a letter for the subclass of either A for alpha or B for beta, according to the suggestion by Klein *et al.* [17]. The first expressed teleost MHC class II beta sequences were published in zebrafish [58] and were later shown to be allelic variants of a single locus defined as *DareDAB* [59,60,61,62]. MHC class II alpha sequences were also first found in zebrafish [63]. Since then, MHC class II alpha and beta sequences have been reported in a wide variety of teleosts where only a few are referenced here [64,65,66,67,68,69].

**Figure 6:**
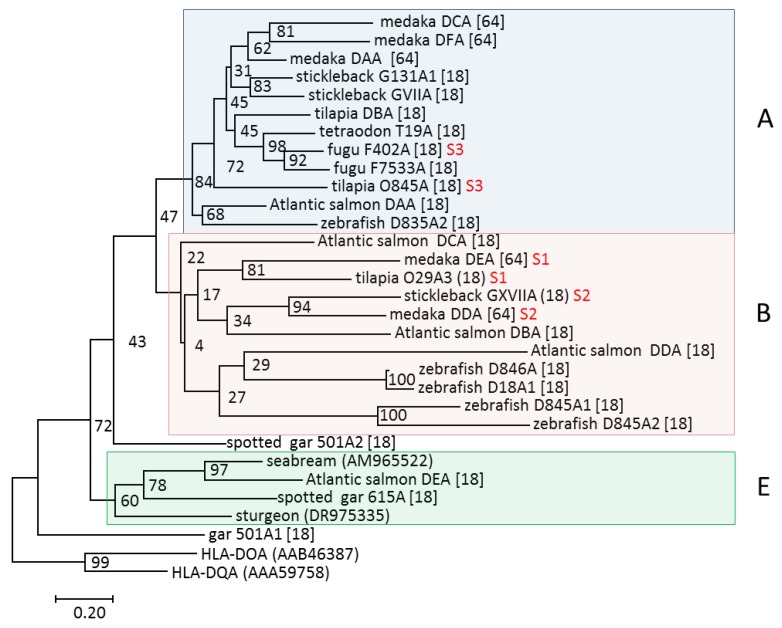
Phylogeny of MHC class II alpha 1 domain sequences modified from Dijkstra *et al.* [18]. Individual lineages are shown on the right hand side of each shaded clade. Neoteleost genes residing within the three syntenic regions S1–S3 are shown using red font. The bootstrap consensus tree was inferred from 1000 replicates, is drawn to scale, and has branch lengths representing the number of amino acid substitutions per site (see scale bar). The tree is rooted using the human MHC class II sequences HLA-DQA and HLA-DOA. GenBank sequence references are in parenthesis while the remaining sequences can be found in [18,64].

The observation that zebrafish MHC class I and class II genes were unlinked was a surprise [59,61]; identical results have also been reported for other teleosts, substantiating this as a general rule for these two classes in teleost [35,37,70,71]. Similar to MHC class I, several lineages have been defined for teleost MHC class II based on phylogenetic clustering [18,72,73]. Bannai and Nonaka proposed a lineage A [64], corresponding to DA lineage in Dijkstra *et al.* [18], and lineages D and E, encompassing sequences within the DB group of Dijkstra *et al.* To resolve this lineage nomenclature confusion, I suggest using the term A for sequences clustering with salmonid and medaka classical MHCII sequences DAA-DAB, while retaining E for sequences clustering with salmonid DEA-DEB sequences. The B group definition can then be used for sequences clustering with Atlantic salmon *DBA-DBB*, *DCA-DCB*, and *DDA-DDB* sequences and medaka *DEA-DEB*, *DDA-DDB1/2* sequences. The A and E lineages display consistent clustering and represent true lineages, while sequences clustering within the B clade display a more variable clustering and may thus not represent a true lineage (Figure 6).

#### 2.2.1. The A Lineage

All published highly-expressed and/or polymorphic teleost MHCII gene sequences belong to the A lineage (Figure 6) [18,64]. These sequences display a high degree of conservation of the alpha 1 domain residues N62 and N69 and the beta 1 domain residues H81 and N82, which in classical mammalian MHCII molecules make important hydrogen bonds with the backbone of peptide ligands. A few A lineage molecules lack these peptide-binding residues and might exert nonclassical functions. Two residues known to be important for CD4 binding in mammalian species, *i.e.*, βS144 and βE162, are also highly conserved within A lineage sequences.

So far we only have sufficient data from salmonids and medaka to decipher between classical and nonclassical MHCII genes [18,64]. In Atlantic salmon, there are only two A lineage genes, denoted *SasaDAA* and *SasaDAB*, encoding the MHCII alpha and beta chains, respectively. These two genes are the only classical MHCII genes in Atlantic salmon and rainbow trout combining high polymorphism, classical expression patterns, and peptide anchoring and CD4 binding capacity [18,72,73]. A total of 22 alleles are defined for the Atlantic salmon *SasaDAA* locus, while 42 alleles are registered for the Atlantic salmon *SasaDAB* locus [1]. The number of registered alleles for rainbow trout is 3 for *OnmyDAA* and 22 for *OnmyDAB*, where the uneven distribution of alpha and beta alleles is probably due to scientific focus rather than a true representation of biological variation. The S*asaDAA* and *SasaDAB* genes are highly expressed in major immune tissues such as gills, gut, head kidney, and spleen, but display much lower transcription levels in non-immune tissues, such as heart, liver, eye, and brain [18]. They also segregate as a functional haplotype due to close linkage on chromosome 12 ([74] and Figure 3). In medaka, there is also only one classical MHCII gene pair, denoted *orlaDAA* and *orlaDAB*, both displaying considerable polymorphism [64]. Two additional medaka gene pair sequences cluster within the A lineage, *i.e.*, *orlaDCA-DCB* and *orlaDFA-DFB*, but these genes are nonpolymorphic and are primarily expressed in gills. Linking polymorphism to individual genes has not been performed in sufficient detail for other species to decipher between classical and nonclassical.

In zebrafish, there are 14 genes belonging to the A lineage. Of these, eleven have been shown to be expressed where three alpha genes and two beta genes display abundant transcription [18]. One of the abundantly expressed MHCII beta genes (D8.37B3, [18]) has been defined as *DareDAB*. Another sequence, defined as *DareDAA* [61], is not found in the zebrafish Ensembl Zv9 genome, potentially suggesting haplotypic variation in number of MHCII genes. Further studies are needed to define the abundantly expressed zebrafish A lineage alpha and beta sequence as classical or nonclassical. The number of A lineage genes in other teleosts ranges from six in medaka to 33 in tilapia [18,73]. Although we know that some of these genes are expressed [18], there is not sufficient data to define classical *versus* nonclassical loci.

Multiple studies have provided evidence for the effect of specific MHC class II alpha and beta haplotypes influencing resistance against the bacterial pathogen *Aeromonas salmonicida* causing furunculosis in Atlantic salmon [75,76,77,78]. Here, some classical alpha and beta haplotypes were found to be significantly associated with resistance, while other haplotypes were found significantly associated with susceptibility towards the disease. Similar disease association studies have also been performed for MHC class I, but these results lack substantiation by different research groups [75,79,80,81,82]. Two of these studies also identified the salmonid nonclassical MHCI region, and not the classical region, underlying the disease associations [80,81].

#### 2.2.2. The B Group

Sequences belonging to the B group and E lineage all comply with a nonclassical definition, with low polymorphism, expression patterns deviating from their classical counterparts, and/or lack of the peptide anchoring and CD4 binding residues. Initially, nonclassical MHCII genes were found by McConnell *et al.* [83], but were first defined as nonclassical genes representing a new lineage by Harstad *et al.* [72]. Sequences belonging to this B group have now been found in salmonids, medaka, tilapia, zebrafish and stickleback [18,64,72,73,83].

The B group consists of at least three sub-groups, defined by sequences clustering with the zebrafish D8.45/D8.46, Atlantic salmon DB, and DC sequences (Figure 6) [18]. The Atlantic salmon DB group molecules are encoded by the genes *SasaDBA*/*-DBB*, *-DCA*/*-DCB1/2*, and -*DDA*/-*DDB,* where the majority of alpha and beta genes are closely linked, similar to the organization of most MHCII genes in teleosts. All Atlantic salmon B group genes are virtually nonpolymorphic and are expressed at low or intermediate levels in all tested tissues, with some tissue-specific distribution patterns [18]. Medaka MHCII sequences clustering within the B group, all of a nonclassical nature, are denoted *orlaDDA-DDB1/2* and *orlaDEA-DEB* [64], where the *orlaDEA-DEB* gene sequences cluster with *sasaDCA-DCB*, while the *orlaDDA-DDB* sequences cluster with *sasaDBA-DBB* (Figure 6). Contrary to what the names indicate, the medaka *DEA-DEB* genes are not orthologous to the salmonid *DEA-DEB* genes. In other investigated teleosts, the number of B group sequences range from none in tetraodon and fugu to 16 in tilapia.

Expression pattern and phylogeny are not sufficient to ascertain a function for these B group molecules in teleosts, so Dijkstra *et al.* [18] performed a thorough comparison with the mammalian nonclassical MHCII molecules. In humans, the only two nonclassical MHCII molecules are HLA-DM, which provides support for peptide loading and editing of the classical MHCII molecules, and HLA-DO, which regulates HLA-DM function [15,84]. Distinct features of these human nonclassical molecules, such as endosomal sorting motif in the cytoplasmic tail and critical residues providing molecular interactions, are not present in most teleost B group sequences. Also, A lineage molecules do not contain the residue used by tetrapod classical molecules for DM interaction. Thus, teleosts appear not to have replicates of the human HLA-DM and HLA-DO system.

When speculating upon the function of teleost nonclassical MHC class I sequences, there is a diverse array of mammalian molecules and functions to choose from. For MHC class II, the list of human nonclassical molecules is limited to HLA-DM and HLA-DO. Although the features defining classical MHCII and HLA-DM interactive function in tetrapods are not preserved in teleosts, they may have evolved other features for accomplishing the same task. Different teleosts may also have evolved different approaches in editing and modifying MHCII peptide loading, as some species lack B group sequences altogether. The fact that Atlantic salmon DBB and DCB genes, in addition to the stickleback GXVIIB gene, all harbor an endosomal sorting motif in their cytoplasmic domain may hint to HLA-DM function for these sequences in these species. However, this needs future functional evidence to be substantiated.

#### 2.2.3. The E Lineage

E, the third discernable lineage, is found in seabream, fathead minnow, carp, loach, spotted gar, sturgeon, Atlantic salmon, and rainbow trout (Figure 6) [18,85]. The Atlantic salmon *SasaDEA* and *SasaDEB* genes are nonpolymorphic and expressed at very low levels in all tested tissues. Due to the salmonid WGD event 95 million years ago [38], a duplicate region containing pseudogenes resides on chromosome 5 as opposed to the S*asaDEA* and *SasaDEB* genes residing on chromosome 2 (Figure 3). E lineage sequences differ significantly from all other teleost fish MHCII sequences, sharing several features even with tetrapod classical and nonclassical class II molecules [18]. The similarity with tetrapod MHCII sequences is also reflected in the genomic organization of salmonid E lineage genes sharing many of the genes residing within the human MHC region (see Section 2.4). The functional relevance of E lineage genes is unknown, but they may have been replaced by more successful A/lineage molecules.

### 2.3. MHC and Deeper Phylogenies

**Figure 7:**
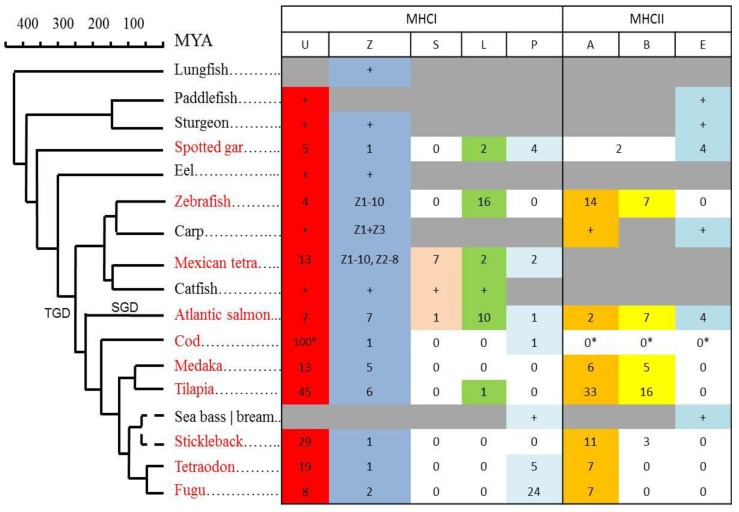
Phylogenetic distributions and gene count for MHC class I and class II lineages. Presence of MHCI (U,Z,S,L,P; data from [19]) and MHCII (A,B,E; data from [18]) lineages are shown in a tabular format linked to the phylogeny of each individual species. Species with sequenced genomes that have been studied in detail are shown using red font. Number of genes within each lineage are shown, where the “+” refers to at least one sequence identified in species without sequenced genomes. Presence of a lineage is marked using individual colors. Lineages not identified are shown using white cells labelled 0, where the MHCII data for cod originates from Star *et al.*(* [33]). Lineages we have not looked for or there is insufficient data to conclude are shown using grey cells. Number of Z1–Z3 lineage genes are shown for zebrafish and Mexican tetra, but unknown for carp, lacking sequenced genomes. For spotted gar, two MHCII sequences could not be assigned as A or B and are thus shown as A/B [18]. The P lineage was found in sea bass, while the E lineage was found in sea bream.

For MHC class I, all studied teleosts have both U and Z lineage genes, although the number of genes varies between 7–100 U lineage genes and 1–18 Z lineage genes [19]. Both U and Z lineage molecules most likely bind peptides, but only the U lineage contains molecules of a classical nature with extensive polymorphism. The U lineage can be traced back to a common ancestor of paddlefish and sturgeon (Figure 7), although in primitive bony fish the U lineage characteristics become more diffuse.

The Z lineage has members in spotted gar, sturgeon, and paddlefish, and can be traced as far back as lungfish. This lineage has thus coexisted alongside classical type molecules for more than 450 million years. Based on the almost complete conservation of its peptide binding residues, the as yet unknown function of this sub-lineage has also been preserved. The remaining MHCI lineages L, S, and P do not display the characteristic peptide-binding motif, and most likely either bind other ligands or no ligands. The L and P lineages can be traced back to spotted gar, while the S lineage has not been found further back than Ostariophysi, exemplified by Mexican tetra and catfish.

For MHC class II, sturgeon and paddlefish sequences phylogenetically cluster with E lineage sequences, suggesting that the A/B lineage emerged after paddlefish/sturgeon split from the main teleost lineage [18]. This is supported by the fact that in spotted gar, E lineage sequences coexist alongside A/B-like sequences in a region also containing a classical-like MHC class I gene. This is also consistent with the E lineage, having preserved several of the characteristic tetrapod features and also linkage to many of the MHC-region genes also found in the mammalian MHC. The A/B lineage then evolved further into a distinct A lineage and a more diffuse B group.

### 2.4. Regional Syntenies and Functional Haplotypes

**Figure 8:**
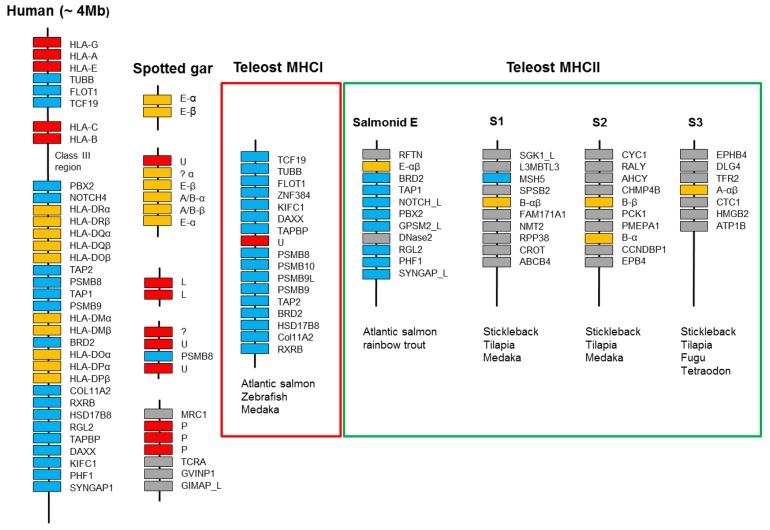
Schematic presentation of selected MHC regions. Red boxes represent MHCI genes, orange boxes MHCII genes, cyan boxes are genes shared with the human MHC region, while grey boxes are genes without such synteny. Only relevant genes within the 4 Mb human MHC region are shown and the region is not drawn to scale. The chromosomal organization of the spotted gar MHC regions is currently unknown, where sequences of undefined MHC lineages are shown using a question mark. Only syntenic genes within the teleost MHCI region present in the three representatives of Ostariophysi (zebrafish), salmonids (Atlantic salmon), and neoteleosts (medaka) are shown. For MHC class II, both lineage/group and type of molecule (alpha or beta) are shown, where the number of class II genes display some variation between species. Synteny regions S1–S3 are the only three syntenic MHCII regions found in some neoteleosts. These regions have lost most of the typical MHC region genes still present in the salmonid E region. Details on the ray-finned MHC regions can be found in [18,19].

The duplicate Atlantic salmon MHCI region, as well as core MHCI regions identified in other teleosts, share many genes with the human MHC region [35,37,70,71,86], where close physical linkage may confer a functional advantage. Genes influencing MHCI peptide loading, such as tapasin (TAPBP), transporter associated protein 2 (TAP2), and the immunoproteasome components PSMB8, PSMB9 and PSMB10 are all part of the core teleost MHC (Figure 8). In addition, genes with less obvious influence on MHCI function are also present, such as retinoic X receptor beta (RXRB), bromodomain containing 2 (BRD2), transcription factor 19 (TCF19), and death-domain associated protein (DAXX). Retinoid X Receptor Beta (RXRB), a member of the nuclear hormone receptor superfamily, may activate transcription of MHC class I genes in response to retinoic acid [87]. BRD2 is known to couple chromatin to cell-cycle progression in addition to promoting B cell expansions, but its effect on MHCI is currently unknown [88]. The functional advantage of having all these genes within the major teleost MHCI region may become apparent in the future.

Other haplotypes with a potential preselected functional advantage have also been published in teleosts. Studies in medaka showed divergent haplotypes of classical MHCI, PSMB8, and PSMB10 alleles, suggesting a functional advantage of having these proteasome subunit variants linked to a given MHCI allele [86]. Zebrafish also harbors potential classical MHCI and divergent PSMB8 haplotypes [28]. Similar examples are also found in other nonfish species, such as chicken and frog. For instance, chicken, representing a minimal essential MHC region of only 92 kb with 19 genes, has one dominantly expressed MHCI locus flanked by polymorphic TAP and tapasin genes segregating as likely functional haplotypes [89,90]. Frog also has presumed functional haplotypes of co-segregating highly divergent MHCI, TAP1, TAP2, and PSMB8 alleles [91].

For MHC class II, the situation is very different, with the majority of teleost MHCII genes being scattered in regions lacking defined synteny [18]. The only exceptions are three regions shared between some neoteleosts (Figure 8, [18]). This lack of synteny implies that the class II has translocated to different locations in various teleosts, in line with the idea that the class II translocated out of a primordial teleost MHC region. The un-linkage of the MHCI and II genes occurred in a teleost ancestor as the two classes are still linked in spotted gar. Remnants of this primordial region are still seen in the salmonid MHCII E region. The Atlantic salmon *SasaDEA* and *SasaDEB* genes on chromosome 2 reside in a typical MHC region containing the genes BRD2, TAP1, NOTCH(-like), PBX2, GPSM2(-like), RGL2, PHF1, and SYNGAP [18]. The duplicated region on chromosome 5 contains a similar set of genes and an orthologous region is also found in rainbow trout [85]. Having also retained many ancestral features with tetrapod classical and nonclassical MHCII sequences, the E lineage represents the primordial ray-finned MHC class II lineage.

## 3. Conclusions

With available genome sequences, we have now likely defined all the various MHC lineages in teleosts. For MHC class I, five distinct lineages have been defined through phylogenetic studies. The U and typical Z lineages, which are present in all studied species, already coexisted in primitive ray-finned fishes. This coexistence of the typical Z lineage alongside a classical-like sequence, where U lineage characteristics are more diffuse, can also be found in lungfish, a stem sarcopterygian species. Classical MHCI sequences all belong to the U lineage, while the typical Z lineage molecule likely binds a highly similar or identical peptide as yet unknown ligand in all studied species. The remaining MHCI lineages, S, L, and P, presumably do not bind peptides and are not present in all studied species.

For MHC class II, three teleost “lineages” have been defined through phylogenetic studies. The E lineage is present in the primitive ray-finned fish sturgeon and paddlefish. In spotted gar, the E lineage resides closely linked to A/B-like gene sequences as well as MHC class I, representing the ancient MHC gene organization found in shark and tetrapods. The A/B lineage has since multiplied and diversified into A and B lineages in teleosts where the A lineage is present in all studied species with the exception of gadoids, while the E and B lineages are not. Classical MHCII genes all belong to the A lineage and have been proven to influence disease resistance in some species.

For both MHC classes, the fact that some lineages are absent in many species raises some concern regarding their functional relevance. Perhaps different species have evolved different approaches to handling aspects such as MHCII peptide loading and editing where some use chaperones and others do not. Teleosts are the most species-rich and diversified group of all the vertebrates, which may have provided them with ample time to evolve different approaches to antigen presentation. Thus, the future still holds promise for many intriguing discoveries in the field of teleost MHC.

## References

[1] IPD-MHC Database. http://www.ebi.ac.uk/ipd/mhc/fish/.

[2] Lee N., Goodlett D.R., Ishitani A., Marquardt H., Geraghty D.E. (1998). HLA-E surface expression depends on binding of TAP-dependent peptides derived from certain HLA class I signal sequences. J. Immunol..

[3] Lee N., Llano M., Carretero M., Ishitani A., Navarro F., Lopez-Botet M., Geraghty D.E. (1998). HLA-E is a major ligand for the natural killer inhibitory receptor CD94/NKG2A. Proc. Natl. Acad. Sci. USA.

[4] Pietra G., Romagnani C., Manzini C., Moretta L., Mingari M.C. (2010). The emerging role of HLA-E-restricted CD8+ T lymphocytes in the adaptive immune response to pathogens and tumors. J. Biomed. Biotechnol..

[5] Goodridge J.P., Burian A., Lee N., Geraghty D.E. (2010). HLA-F complex without peptide binds to MHC class I protein in the open conformer form. J. Immunol..

[6] Goodridge J.P., Burian A., Lee N., Geraghty D.E. (2013). HLA-F and MHC class I open conformers are ligands for NK cell Ig-like receptors. J. Immunol..

[7] Goodridge J.P., Lee N., Burian A., Pyo C.W., Tykodi S.S., Warren E.H., Yee C., Riddell S.R., Geraghty D.E. (2013). HLA-F and MHC-I open conformers cooperate in a MHC-I antigen cross-presentation pathway. J. Immunol..

[8] Adams E.J., Luoma A.M. (2013). The adaptable major histocompatibility complex (MHC) fold: Structure and function of nonclassical and MHC class I-like molecules. Annu. Rev. Immunol..

[9] De Libero G., Mori L. (2010). How the immune system detects lipid antigens. Prog. Lipid Res..

[10] Facciotti F., Cavallari M., Angenieux C., Garcia-Alles L.F., Signorino-Gelo F., Angman L., Gilleron M., Prandi J., Puzo G., Panza L. (2011). Fine tuning by human CD1e of lipid-specific immune responses. Proc. Natl. Acad. Sci. USA.

[11] Moody D.B., Porcelli S.A. (2001). CD1 trafficking: Invariant chain gives a new twist to the tale. Immunity.

[12] Montes R., Puy C., Molina E., Hermida J. (2012). Is EPCR a multi-ligand receptor? Pros and cons. Thromb. Haemost..

[13] Kennedy M.W., Heikema A.P., Cooper A., Bjorkman P.J., Sanchez L.M. (2001). Hydrophobic ligand binding by Zn-alpha 2-glycoprotein, a soluble fat-depleting factor related to major histocompatibility complex proteins. J. Biol. Chem..

[14] Kjer-Nielsen L., Patel O., Corbett A.J., Le Nours J., Meehan B., Liu L., Bhati M., Chen Z., Kostenko L., Reantragoon R. (2012). MR1 presents microbial vitamin B metabolites to MAIT cells. Nature.

[15] Neefjes J., Jongsma M.L., Paul P., Bakke O. (2011). Towards a systems understanding of MHC class I and MHC class II antigen presentation. Nat. Rev. Immunol..

[16] Hashimoto K., Nakanishi T., Kurosawa Y. (1990). Isolation of carp genes encoding major histocompatibility complex antigens. Proc. Natl. Acad. Sci. USA.

[17] Klein J., Bontrop R.E., Dawkins R.L., Erlich H.A., Gyllensten U.B., Heise E.R., Jones P.P., Parham P., Wakeland E.K., Watkins D.I. (1990). Nomenclature for the major histocompatibility complexes of different species: a proposal. Immunogenetics.

[18] Dijkstra J.M., Grimholt U., Leong J., Koop B.F., Hashimoto K. (2013). Comprehensive analysis of MHC class II genes in teleost fish genomes reveals dispensability of the peptide-loading DM system in a large part of vertebrates. BMC Evol. Biol..

[19] Grimholt U., Tsukamoto K., Azuma T., Leong J., Koop B.F., Dijkstra J.M. (2015). A comprehensive analysis of teleost MHC class I sequences. BMC Evol. Biol..

[20] Grimholt U., Hordvik I., Fosse V.M., Olsaker I., Endresen C., Lie O. (1993). Molecular cloning of major histocompatibility complex class I cDNAs from Atlantic salmon (*Salmo salar*). Immunogenetics.

[21] Grimholt U., Drablos F., Jorgensen S.M., Hoyheim B., Stet R.J. (2002). The major histocompatibility class I locus in Atlantic salmon (*Salmo salar* L.): Polymorphism, linkage analysis and protein modelling. Immunogenetics.

[22] Nonaka M.I., Nonaka M. (2010). Evolutionary analysis of two classical MHC class I loci of the medaka fish, *Oryzias latipes*: Haplotype-specific genomic diversity, locus-specific polymorphisms, and interlocus homogenization. Immunogenetics.

[23] Hansen J.D., Strassburger P., Du P.L. (1996). Conservation of an alpha 2 domain within the teleostean world, MHC class I from the rainbow trout *Oncorhynchus mykiss*. Dev. Comp. Immunol..

[24] Katagiri T., Hirono I., Aoki T., Sakai M. (1996). Isolation of major histocompatibility complex class I cDNA from pink salmon (*Oncorhynchus gorbuscha*). Dev. Comp. Immunol..

[25] Aoyagi K., Dijkstra J.M., Xia C., Denda I., Ototake M., Hashimoto K., Nakanishi T. (2002). Classical MHC class I genes composed of highly divergent sequence lineages share a single locus in rainbow trout (*Oncorhynchus mykiss*). J. Immunol..

[26] Michalova V., Murray B.W., Sultmann H., Klein J. (2000). A contig map of the MHC class I genomic region in the zebrafish reveals ancient synteny. J. Immunol..

[27] Takeutchi H., Figueroa F., O’hUigin C., Klein J. (1995). Cloning and characterization of class I Mhc genes of the zebrafish, *Brachydanio rerio*. Immunogenetics.

[28] McConnell S.C., Restaino A.C., de Jong J.L. (2014). Multiple divergent haplotypes express completely distinct sets of class I MHC genes in zebrafish. Immunogenetics.

[29] Mehta R.B., Nonaka M.I., Nonaka M. (2009). Comparative genomic analysis of the major histocompatibility complex class I region in the teleost genus Oryzias. Immunogenetics.

[30] Kiryu I., Dijkstra J.M., Sarder R.I., Fujiwara A., Yoshiura Y., Ototake M. (2005). New MHC class Ia domain lineages in rainbow trout (*Oncorhynchus mykiss*) which are shared with other fish species. Fish Shellfish Immunol..

[31] Nonaka M.I., Aizawa K., Mitani H., Bannai H.P., Nonaka M. (2011). Retained orthologous relationships of the MHC Class I genes during euteleost evolution. Mol. Biol. Evol..

[32] Shum B.P., Guethlein L., Flodin L.R., Adkison M.A., Hedrick R.P., Nehring R.B., Stet R.J., Secombes C., Parham P. (2001). Modes of salmonid MHC class I and II evolution differ from the primate paradigm. J. Iimmunol..

[33] Star B., Nederbragt A.J., Jentoft S., Grimholt U., Malmstrom M., Gregers T.F., Rounge T.B., Paulsen J., Solbakken M.H., Sharma A. (2011). The genome sequence of Atlantic cod reveals a unique immune system. Nature.

[34] Miller K.M., Li S., Ming T.J., Kaukinen K.H., Schulze A.D. (2006). The salmonid MHC class I: More ancient loci uncovered. Immunogenetics.

[35] Lukacs M.F., Harstad H., Bakke H.G., Beetz-Sargent M., McKinnel L., Lubieniecki K.P., Koop B.F., Grimholt U. (2010). Comprehensive analysis of MHC class I genes from the U-, S-, and Z-lineages in Atlantic salmon. BMC genomics.

[36] Lukacs M.F., Harstad H., Grimholt U., Beetz-Sargent M., Cooper G.A., Reid L., Bakke H.G., Phillips R.B., Miller K.M., Davidson W.S. (2007). Genomic organization of duplicated major histocompatibility complex class I regions in Atlantic salmon (*Salmo salar*). BMC genomics.

[37] Shiina T., Dijkstra J.M., Shimizu S., Watanabe A., Yanagiya K., Kiryu I., Fujiwara A., Nishida-Umehara C., Kaba Y., Hirono I. (2005). Interchromosomal duplication of major histocompatibility complex class I regions in rainbow trout (*Oncorhynchus mykiss*), a species with a presumably recent tetraploid ancestry. Immunogenetics.

[38] Macqueen D.J., Johnston I.A. (2014). A well-constrained estimate for the timing of the salmonid whole genome duplication reveals major decoupling from species diversification. Proc. Biol. Sci..

[39] Lien S., Gidskehaug L., Moen T., Hayes B.J., Berg P.R., Davidson W.S., Omholt S.W., Kent M.P. (2011). A dense SNP-based linkage map for Atlantic salmon (*Salmo salar*) reveals extended chromosome homeologies and striking differences in sex-specific recombination patterns. BMC genomics.

[40] Phillips R.B., Keatley K.A., Morasch M.R., Ventura A.B., Lubieniecki K.P., Koop B.F., Danzmann R.G., Davidson W.S. (2009). Assignment of Atlantic salmon (*Salmo salar*) linkage groups to specific chromosomes: Conservation of large syntenic blocks corresponding to whole chromosome arms in rainbow trout (*Oncorhynchus mykiss*). BMC Genet..

[41] Dijkstra J.M., Kiryu I., Yoshiura Y., Kumanovics A., Kohara M., Hayashi N., Ototake M. (2006). Polymorphism of two very similar MHC class Ib loci in rainbow trout (*Oncorhynchus mykiss*). Immunogenetics.

[42] Miller K.M., Kaukinen K.H., Schulze A.D. (2002). Expansion and contraction of major histocompatibility complex genes: A teleostean example. Immunogenetics.

[43] Sato A., Dongak R., Hao L., Takezaki N., Shintani S., Aoki T., Klein J. (2006). Mhc class I genes of the cichlid fish *Oreochromis niloticus*. Immunogenetics.

[44] Okamura K., Nakanishi T., Kurosawa Y., Hashimoto K. (1993). Expansion of genes that encode MHC class I molecules in cyprinid fish. J. Immunol..

[45] Kruiswijk C.P., Hermsen T.T., Westphal A.H., Savelkoul H.F., Stet R.J. (2002). A novel functional class I lineage in zebrafish (*Danio rerio*), carp (*Cyprinus carpio*), and large barbus (*Barbus intermedius*) showing an unusual conservation of the peptide binding domains. J. Immunol..

[46] Stet R.J., Kruiswijk C.P., Dixon B. (2003). Major histocompatibility lineages and immune gene function in teleost fishes: The road not taken. Crit. Rev. Immunol..

[47] Stet R.J., Kruiswijk C.P., Saeij J.P., Wiegertjes G.F. (1998). Major histocompatibility genes in cyprinid fishes: theory and practice. Immunol. Rev..

[48] Hashimoto K., Okamura K., Yamaguchi H., Ototake M., Nakanishi T., Kurosawa Y. (1999). Conservation and diversification of MHC class I and its related molecules in vertebrates. Immunol. Rev..

[49] Saper M.A., Bjorkman P.J., Wiley D.C. (1991). Refined structure of the human histocompatibility antigen HLA-A2 at 2.6 A resolution. J. Mol. Biol..

[50] Dirscherl H., Yoder J.A. (2013). Characterization of the Z lineage Major histocompatability complex class I genes in zebrafish. Immunogenetics.

[51] Shum B.P., Rajalingam R., Magor K.E., Azumi K., Carr W.H., Dixon B., Stet R.J., Adkison M.A., Hedrick R.P., Parham P. (1999). A divergent non-classical class I gene conserved in salmonids. Immunogenetics.

[52] Campbell E.C., Antoniou A.N., Powis S.J. (2012). The multi-faceted nature of HLA class I dimer molecules. Immunology.

[53] Dijkstra J.M., Katagiri T., Hosomichi K., Yanagiya K., Inoko H., Ototake M., Aoki T., Hashimoto K., Shiina T. (2007). A third broad lineage of major histocompatibility complex (MHC) class I in teleost fish; MHC class II linkage and processed genes. Immunogenetics.

[54] Dirscherl H., McConnell S.C., Yoder J.A., de Jong J.L. (2014). The MHC class I genes of zebrafish. Dev. Comp. Immunol..

[55] Elmer B.M., McAllister A.K. (2012). Major histocompatibility complex class I proteins in brain development and plasticity. Trends Neurosci..

[56] Huang Y.H., Airas L., Schwab N., Wiendl H. (2011). Janus head: The dual role of HLA-G in CNS immunity. Cell. Mol. Life Sci..

[57] Renthal N.E., Guidry P.A., Shanmuganad S., Renthal W., Stroynowski I. (2011). Isoforms of the nonclassical class I MHC antigen H2-Q5 are enriched in brain and encode Qdm peptide. Immunogenetics.

[58] Ono H., Klein D., Vincek V., Figueroa F., O’hUigin C., Tichy H., Klein J. (1992). Major histocompatibility complex class II genes of zebrafish. Proc. Natl. Acad. Sci. USA.

[59] Bingulac-Popovic J., Figueroa F., Sato A., Talbot W.S., Johnson S.L., Gates M., Postlethwait J.H., Klein J. (1997). Mapping of MHC class I and class II regions to different linkage groups in the zebrafish, *Danio rerio*. Immunogenetics.

[60] Graser R., Vincek V., Takami K., Klein J. (1998). Analysis of zebrafish Mhc using BAC clones. Immunogenetics.

[61] Kuroda N., Figueroa F., O’HUigin C., Klein J. (2002). Evidence that the separation of Mhc class II from class I loci in the zebrafish, *Danio rerio*, occurred by translocation. Immunogenetics.

[62] Sultmann H., Mayer W.E., Figueroa F., O’Huigin C., Klein J. (1994). Organization of Mhc class II B genes in the zebrafish (*Brachydanio rerio*). Genomics.

[63] Sultmann H., Meyer W.E., Figueroa F., O’hUigin C., Klein J. (1993). Zebrafish Mhc class II alpha chain-encoding genes: Polymorphism, expression and function. Immunogenetics.

[64] Bannai H.P., Nonaka M. (2013). Comprehensive analysis of medaka major histocompatibility complex (MHC) class II genes: Implications for evolution in teleosts. Immunogenetics.

[65] Godwin U.B., Antao A., Wilson M.R., Chinchar V.G., Miller N.W., Clem L.W., McConnell T.J. (1997). MHC class II B genes in the channel catfish (*Ictalurus punctatus*). Dev. Comp. Immunol..

[66] Hordvik I., Grimholt U., Fosse V.M., Lie O., Endresen C. (1993). Cloning and sequence analysis of cDNAs encoding the MHC class II beta chain in Atlantic salmon (*Salmo salar*). Immunogenetics.

[67] Miller K.M., Withler R.E. (1996). Sequence analysis of a polymorphic Mhc class II gene in Pacific salmon. Immunogenetics.

[68] Ono H., O’hUigin C., Vincek V., Stet R.J.M., Figueroa F., Klein J. (1993). New beta-chain encoding Mhc class II genes in the carp. Immunogenetics.

[69] Sato A., Figueroa F., O’HUigin C., Steck N., Klein J. (1998). Cloning of major histocompatibility complex (Mhc) genes from threespine stickleback, *Gasterosteus aculeatus*. Mol. Mar. Biol. Biotech..

[70] Clark M.S., Shaw L., Kelly A., Snell P., Elgar G. (2001). Characterization of the MHC class I region of the Japanese pufferfish (*Fugu rubripes*). Immunogenetics.

[71] Matsuo M., Asakawa S., Shimizu N., Kimura H., Nonaka M. (2002). Nucleotide sequence of the MHC class I genomic region of a teleost, the medaka (*Oryzias latipes*). Immunogenetics.

[72] Harstad H., Lukacs M.F., Bakke H.G., Grimholt U. (2008). Multiple expressed MHC class II loci in salmonids; details of one non-classical region in Atlantic salmon (*Salmo salar*). BMC genomics.

[73] Sato A., Dongak R., Hao L., Shintani S., Sato T. (2012). Organization of Mhc class II A and B genes in the tilapiine fish Oreochromis. Immunogenetics.

[74] Stet R.J., de Vries B., Mudde K., Hermsen T., van Heerwaarden J., Shum B.P., Grimholt U. (2002). Unique haplotypes of co-segregating major histocompatibility class II A and class II B alleles in Atlantic salmon (*Salmo salar*) give rise to diverse class II genotypes. Immunogenetics.

[75] Grimholt U., Larsen S., Nordmo R., Midtlyng P., Kjoeglum S., Storset A., Saebo S., Stet R.J. (2003). MHC polymorphism and disease resistance in Atlantic salmon (*Salmo salar*); facing pathogens with single expressed major histocompatibility class I and class II loci. Immunogenetics.

[76] Kjoglum S., Larsen S., Grimholt U. (2008). The effect of specific MHC class I and class II allele combinations on resistance to furunculosis in Atlantic salmon (*Salmo salar*). Scand. J. Immunol..

[77] Langefors A., Lohm J., Grahn M., Andersen O., von Schantz T. (2001). Association between major histocompatibility complex class IIB alleles and resistance to *Aeromonas salmonicida* in Atlantic salmon. Proc. Biol. Sci. / R. Soc..

[78] Lohm J., Grahn M., Langefors A., Andersen O., Storset A., von Schantz T. (2002). Experimental evidence for major histocompatibility complex-allele-specific resistance to a bacterial infection. Proc. Biol. Sci. / R. Soc..

[79] Kjoglum S., Larsen S., Bakke H.G., Grimholt U. (2006). How specific MHC class I and class II combinations affect disease resistance against infectious salmon anaemia in Atlantic salmon (*Salmo salar*). Fish Shellfish Immunol..

[80] Miller K.M., Winton J., Schulze A., Purcell M., Ming T. (2004). Major Histocompatibility Complex Loci are Associated with Susceptibility of Atlantic Salmon to Infectious Hematopoietic Necrosis Virus. Environ. Biol. Fish..

[81] Ozaki A., Khoo S.K., Yoshiura Y., Ototake M., Sakamoto T., Dijkstra J.M., Okamoto N. (2007). Identification of Additional Quantitative Trait Loci (QTL) Responsible for Susceptibility to Infectious Pancreatic Necrosis Virus in Rainbow Trout. Fish Pathol..

[82] Ozaki A., Sakamoto T., Khoo S., Nakamura K., Coimbra M.R., Akutsu T., Okamoto N. (2001). Quantitative trait loci (QTLs) associated with resistance/susceptibility to infectious pancreatic necrosis virus (IPNV) in rainbow trout (*Oncorhynchus mykiss*). Mol.Genet.Genomics.

[83] McConnell T.J., Godwin U., Cuthbertson B.J. (1998). Expressed major histocompatibility complex class II loci in fishes. Immunol. Rev..

[84] Guce A.I., Mortimer S.E., Yoon T., Painter C.A., Jiang W., Mellins E.D., Stern L.J. (2013). HLA-DO acts as a substrate mimic to inhibit HLA-DM by a competitive mechanism. Nat. Struct. Mol. Biol..

[85] Palti Y., Rodriguez M.F., Gahr S.A., Hansen J.D. (2007). Evolutionary history of the ABCB2 genomic region in teleosts. Dev Comp. Immunol..

[86] Tsukamoto K., Hayashi S., Matsuo M., Nonaka M., Kondo M., Shima M.I., Asakawa S., Shimizu N., Nonaka M. (2005). Unprecedented intraspecific diversity of the MHC class I region of a teleost medaka, *Oryzias latipes*. Immunogenetics.

[87] Segars J.H., Nagata T., Bours V., Medin J.A., Franzoso G., Blanco J.C., Drew P.D., Becker K.G., An J., Tang T. (1993). Retinoic acid induction of major histocompatibility complex class I genes in NTera-2 embryonal carcinoma cells involves induction of NF-kappa B (p50-p65) and retinoic acid receptor beta-retinoid X receptor beta heterodimers. Mol. Cell. Biol..

[88] Belkina A.C., Blanton W.P., Nikolajczyk B.S., Denis G.V. (2014). The double bromodomain protein Brd2 promotes B cell expansion and mitogenesis. J. Leukoc. Biol..

[89] Kaufman J. (2015). Co-evolution with chicken class I genes. Immunol. Rev..

[90] van Hateren A., Carter R., Bailey A., Kontouli N., Williams A.P., Kaufman J., Elliott T. (2013). A mechanistic basis for the co-evolution of chicken tapasin and major histocompatibility complex class I (MHC I) proteins. J. Biol. Chem..

[91] Ohta Y., Powis S.J., Lohr R.L., Nonaka M., Pasquier L.D., Flajnik M.F. (2003). Two highly divergent ancient allelic lineages of the transporter associated with antigen processing (TAP) gene in Xenopus: Further evidence for co-evolution among MHC class I region genes. Eur. J. Immunol..

